# Measurement of heart rate variability using off-the-shelf smart phones

**DOI:** 10.1186/s12938-016-0127-8

**Published:** 2016-01-29

**Authors:** Ren-You Huang, Lan-Rong Dung

**Affiliations:** Institute of Electrical Control Engineering, National Chiao Tung University, 1001 Ta Hsueh Rd., Hsinchu, Taiwan; Department of Electrical and Computer Engineering, National Chiao Tung University, 1001 Ta Hsueh Rd., Hsinchu, Taiwan

**Keywords:** Cardiac physiology, Non-contact photoplethysmography, Blood volume pulse, Heart rate variability

## Abstract

**Background:**

The cardiac parameters, such as heart rate (HR) and heart rate variability (HRV), are very important physiological data for daily healthcare. Recently, the camera-based photoplethysmography techniques have been proposed for HR measurement. These techniques allow us to estimate the HR contactlessly with low-cost camera. However, the previous works showed limit success for estimating HRV because the R–R intervals, the primary data for HRV calculation, are sensitive to noise and artifacts.

**Methods:**

This paper proposed a non-contact method to extract the blood volume pulse signal using a chrominance-based method followed by a proposed CWT-based denoising technique. The R–R intervals can then be obtained by finding the peaks in the denoised signal. In this paper, we taped 12 video clips using the frontal camera of a smart phone with different scenarios to make comparisons among our method and the other alternatives using the absolute errors between the estimated HRV metrics and the ones obtained by an ECG-accurate chest band.

**Results:**

As shown in experiments, our algorithm can greatly reduce absolute errors of HRV metrics comparing with the related works using RGB color signals. The mean of absolute errors of HRV metrics from our method is only 3.53 ms for the static-subject video clips.

**Conclusions:**

The proposed camera-based method is able to produce reliable HRV metrics which are close to the ones measured by contact devices under different conditions. Thus, our method can be used for remote health monitoring in a convenient and comfortable way.

## Background

Heart rate variability (HRV) provides useful physiological parameters according to the beat-to-beat intervals (R–R intervals, RRI) obtained from cardiac pulse signals. Some publications have validated that abnormality of HRV are related to some cardiological and noncardiological diseases, e.g., myocardial infarction, diabetic neuropathy, and myocardial dysfunction [1]. The conventional way to obtain the cardiac pulse is using electrocardiography (ECG) to sense the electrical activity of heart over a period of time by electrodes attached to the surface of human skin. ECG provides clean and accurate pulse signals, however, it is prone to be interfered by electrical activity produced by skeletal muscles near the electrodes.

Another way to obtain the cardiac pulse is photoplethysmography (PPG), which was first described in 1930s [2]. PPG detects the optical absorption variations of the human skin due to the blood volume variations. Both ECG and PPG need to contact the human skin, which are not suitable for the cases of extreme sensitivity, e.g., neonates, skindamaged patients, or when the non-contact property is required (surveillance, fitness, etc.). Peng et al. [3] proposed an alternative method for extracting the PPG signal through the smart-phone camera followed by computing the HRV. However, this method still requires the subjects to put their finger on the smart-phone camera and keep themselves static, which has similar disadvantages as the traditional PPG device. Recent works have shown that cardiac pulse rate can be measured in a non-contact way, which is also known as remote-PPG (rPPG) [4–12]. These works obtain pulse signals under ambient light conditions with only one camera, which are low-cost, simple, and effective.

The main idea of rPPG is that the blood volume variations can be captured during video recording. The earlier works [4, 5] first obtain the mean intensity of skin region and perform frequency analysis (Fourier or wavelet transform) to estimate the pulse rate. Recent works [6–12] estimate the pulse rate using a regular color video camera. The first step of these methods are locating the region of interest by manual selection or automatic face detection, followed by different analysis algorithms to extract the pulse signals, e.g., difference of RGB [6], source separation [7–9], chrominance [10, 12], motion magnification [11].

Poh et al. [7] proposed an algorithm for heart rate (HR) measurement. They first detected the face every frame and extracted the mean RGB color values to form a three-dimensional time series. Then they applied independent component analysis (ICA) [13, 14] to separate the independent sources from these RGB signals which may contain the pulse signal, followed by FFT and select the frequency with maximum amplitude in the spectral of the component which has highest peak as the HR. Later on, the authors proposed a similar method in [8] to extract the R–R intervals by finding the peaks of the pulse signal. The peaks of pulse signal are treated as the R wave of ECG signal, and the peak intervals are treated as R–R intervals. Alternatively, one may apply PCA, as shown in [9], instead of ICA to separate the pulse signal from RGB time series. Wu et al. [11] proposed an Eulerian-based motion magnification to magnify the subtle motions or color changes in temporal domain using Laplacian pyramid. This method is able to obtain a clean pulse signal if the subjects are almost static. Haan and Jeanne [10] proposed a chrominance-based remote PPG (we denote “C-rPPG” in the rest of this paper) which takes different factors into account to form the color model captured by camera. Given the pulsatility as a function of wavelength exhibits a strong peak in green and the dips in red [15, 16]. To exploit this fact and to reduce the specular reflection problem mentioned in [17], they proposed a model using difference of wighted color channels to obtain chrominance signals. This method is robust to different skin-tone and adaptive to non-white illumination. Moreover, the authors showed the impressive results of HR estimation for the scenario with the subjects exercising on stationary bike. Wang et al. [12] proposed an algorithm exploiting the spatial redundancy of image sensor and the idea of chrominance to improve the robustness to motions.

In the indoor scenes, the lighting sources are usually on the top of subjects, i.e., on ceiling. The color intensities or brightness of the skin captured by camera are various at different positions. Different angles between the lighting sources and camera also result in intensity variations. These periodical or non-periodical variations will produce artifacts which severely influence most of the rPPG algorithms. The methods based on source separation [7–9] may separate the artifacts rather than true pulse signal. Nevertheless, the component with the highest spectral amplitude is not necessary to be the true pulse signal. The Eulerian motion magnification [11] requires the subject as stationary as possible; otherwise, the motion in the specific frequency band will be magnified accordingly. Hence the motion magnification is not appropriate for general scenarios. The C-rPPG [10] improved the robustness to motions and has much better performance in HR estimation with non-static subjects. However, we found that there exist noises and artifacts in the C-rPPG signal which produce false peaks and severely influence the accuracy of R–R intervals. The paper [12] further improves the motion-robustness of the C-rPPG algorithm by adaptively combining local PPG-signals, and improves the SNR using an adaptive band-pass filter. This more elaborated C-rPPG concept, however, leads to an increase in computational complexity, which we consider less attractive for a mobile platform.

This paper proposed a non-contact method to estimate accurate HRV metrics from 30 fps video clips captured by frontal camera of off-the-shelf smart phones. The face of subject is located every frame followed by averaging all the skin pixels to form the RGB time series. The RGB time series are then used to compute the pulse signal by C-rPPG [10] algorithm. We proposed a denoising method based on continuous wavelet transform (CWT) to increase the robustness to interferences. The R–R intervals are obtained by computing the intervals of successive peaks in the denoised signal. To demonstrate the performance of HRV measurement, we taped a 12 video clips for different scenarios with static and non-static subjects and used an ECG-accurate chest band to obtain the R–R intervals as the ground truth. Comparing with existed approaches, the absolute errors of HRV metrics generated by proposed approach is relatively low. For the video clips with static subjects, the mean of absolute errors of the HRV metrics obtained by our method is only 3.53 ms.

## Methods

### Overview

**Fig. 1:**
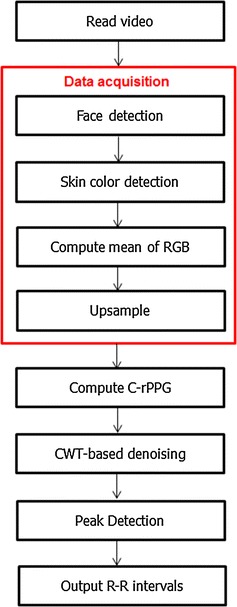
The processing flow of the proposed algorithm

Figure 1 shows the overall flow chart of the proposed algorithm. For each frame of the video, we first detect the face based on the nose positions to increase the robustness to non-frontal faces. Once the face is located, we perform skin detection in YCbCr color space followed by averaged the RGB channels of the skin pixels in the face region to form the time series. To increase the fineness of time grid, we upsample the time series by a factor of eight, i.e., the sampling rate is from 30 to 240 Hz. After data acquisition stage, we compute the C-rPPG signal to extract the pulse signal. Next, we perform our denoising technique based on CWT. Finally, the peaks in the denoised signal are detected to compute the R–R intervals.

### Data acquisition

First of all, we locate the face in every frame to extract the color signals. There are plenty of face detection works and surveys [18–21]. For simplicity and convenience, one may apply the face detector proposed by Viola and Jones [18] which is effective and efficient to locate the faces in frames. However, the face detector will fail if the faces in video are non-frontal. We found that detecting the nose is more stable than detecting the face, thus we can exploit the nose position to derive appropriate face region. We use the object detection toolbox (*vision.CascadeObjectDetector*) built in MATLAB to detect the nose in every frame. The region of the face can be determined by

1 $$\eqalign{ & {w_f}{\text{ }} = {\text{ }}2{w_n} \cr & {h_f}{\text{ }} = {\text{ }}3{h_n} \cr & {x_f}{\text{ }} = {\text{ }}{x_n}{\text{ }} + {\text{ }}{{{w_n}} \mathord{\left/ {\vphantom {{{w_n}} 2}} \right. \kern-\nulldelimiterspace} 2} - {{{w_f}} \mathord{\left/ {\vphantom {{{w_f}} 2}} \right. \kern-\nulldelimiterspace} 2} \cr & {y_f}{\text{ }} = {\text{ }}{y_n}{\text{ }} + {\text{ }}{{{h_n}} \mathord{\left/ {\vphantom {{{h_n}} 2}} \right. \kern-\nulldelimiterspace} 2} - {{{h_f}} \mathord{\left/ {\vphantom {{{h_f}} {1.5}}} \right. \kern-\nulldelimiterspace} {1.5}} \cr}$$

where *w* and *h* are the width and height, (*x*, *y*) is the top left coordinate of the bounding box. The subscripts *n* and *f* represent the “nose” and “face”.

Next, we use a simple skin color detection to ensure the processed data are obtained from skin pixels. There are lots of works one may refer for the skin detection, e.g., the method proposed in [22]. We only take into account the Cb and Cr components to detect the skin color. A pixel is classified as skin pixel if it satisfies the following conditions:

2 $$\begin{aligned} \begin{array}{l} 98 \le Cb \le 142 \\ 133 \le Cr \le 177 \end{array} \end{aligned}$$

After skin detection, we then record the averaged RGB values of skin pixels in the ROI to form the time series. Finally, we upscale the time series by a factor of 8.

### Computing C-rPPG

Inspired by previous works, we apply the chrominance-based method in our algorithm due to the better performance for extracting the real pulse signals instead of false ones. We apply the model $X_smin\alpha Y_s$ proposed in [10] which is briefly reviewed in the following.

In [10], the intensity of a given pixel in *i*-th frame in color channel $C \in \{R,G,B\}$ registered by the camera is modeled as

3 $$\begin{aligned} C_i = I_{C_i}(\rho _{C_{dc}} + \rho _{C_i} + s_i) \end{aligned}$$

where $I_{C_i}$ is the intensity of the light source integrated over the exposure time of the camera, $\rho _{C_{dc}}$ is the stationary part of the reflection coefficient of the skin, $\rho _{C_i}$ is the zero-mean time-varying fraction caused by the pulsation of the blood volume, and $s_i$ is the additive specular reflection contribution.

The RGB data are normalized using the following formula

4 $$\begin{aligned} C_{ni} = \frac{C_i}{\mu (C_i)}, C \in \{R,G,B\} \end{aligned}$$

where $\mu (C_i)$ is a moving average centered around frame index *i*. The chrominance signals are defined as follows

5 $$\begin{aligned} \begin{array}{l} X_s = 3R_n-2G_n \\ Y_s = 1.5R_n + G_n - 1.5B_n \end{array} \end{aligned}$$

Finally, the pulse signals can be extracted by

6 $$\begin{aligned} S = X_f - \alpha Y_f \end{aligned}$$

with

7 $$\begin{aligned} \alpha = \frac{\sigma (X_f)}{\sigma (Y_f)} \end{aligned}$$

where $\sigma (\cdot )$ is standard deviation of the signals, the signals with subscript *f* represent their band-pass filtered versions. We can further rewrite (6) as follows

8 $$\begin{aligned} S = 3(1-\frac{\alpha }{2})R_f - 2(1+\frac{\alpha }{2})G_f + \frac{3\alpha }{2}B_f \end{aligned}$$

### CWT-based denoising method

The CWT transforms a time series to a time-frequency representation and has been used to denoise the PPG signals in some works [23–25]. The CWT uses inner product to measure the similarity between a signal and a specific analysis function, which outperforms the Fourier Transform and the short term Fourier Transform since the CWT can detect rapid changes in frequency due to the multi-scale representation. We will briefly review the theory of CWT and describe our denoising method based on CWT in the following.

The CWT convolves a signal *x*(*t*) with child wavelets $\psi _{\tau ,s}(t)$ which represent scaled and translated versions of mother wavelet $\psi (t)$,

9 $$\begin{aligned} X_w(\tau ,s) = \int _{-\infty }^{\infty } x(t) \psi _{\tau ,s}(t) dt \end{aligned}$$

$X_w(\tau ,s)$ represents similarity between the signal *x*(*t*) and a child wavelet scaled by *s* and translated by $\tau$, which is define as follows:

10 $$\begin{aligned} \psi _{\tau ,s}(t) = \frac{1}{\sqrt{|s|}}\psi \left( \frac{t-\tau }{s} \right) \end{aligned}$$

There are many standard mother wavelets available in CWT literature. We selected the Morlet wavelet in our algorithm since it has been used to analysis PPG signals in [25]. The signal can be reconstructed from the wavelet transform by the inverse formula of (11).

11 $$\begin{aligned} x(t) = \frac{1}{C_{\psi }} \int _{0}^{\infty } \int _{-\infty }^{\infty } \frac{1}{s^2} X_w(\tau ,s)\frac{1}{\sqrt{|s|}}\psi \left( \frac{t-\tau }{s} \right) d\tau ds \end{aligned}$$

where $C_{\psi }$ is the admissible constant of wavelet transform. Let $\hat{\psi }(\xi )$ denoted as Fourier version of $\psi (t)$, the admissible constant is defined as follows:

12 $$\begin{aligned} C_{\psi } = \int _{0}^{\infty }\frac{|\hat{\psi }(\xi )|}{|\xi |} d\xi < \infty \end{aligned}$$

One may reserve the coefficients of specific scales corresponding to the observed frequency band (0.75, 4) Hz [(45, 240) bpm] and set the others to zero followed by inverse transform, which is equivalent to bandpass filtering (we call it “CWT-BP”). However, the motion artifacts are usually in the same frequency band, hence there will be false peaks produced by motion artifacts in the reconstructed signal.

Assume the pulse signal is the most significant component of the C-rPPG signal, our goal is to select a representative scale to reconstruct the pulse signal. We computed the summation of the magnitude of CWT coefficients in the same scale within a time interval, followed by selecting the scale with maximal value of the summation, i.e.,

13 $$\begin{aligned} s^* = \arg \max _s \sum _{\tau } X_w(\tau , s) \end{aligned}$$

where $s^*$ is the optimal scale to reconstruct the pulse signal. The CWT coefficients belonging to the scale $s^*$ are reserved and the others are set to zero. In practice, we should take into account computation efficiency, thus we divide the CWT coefficients into non-overlapping time intervals with length *T* (seconds) and select the representative scales for every time interval. Another factor we should take into account is the non-stationary property of cardiac activity, hence the value of *T* should be carefully selected. Choosing smaller *T* is able to catch up to variation of cardiac activity but is less robust to the strong interference such as motion artifacts, and vice versa. Here we suggest that one can set *T* in the range of 10–30 (seconds). After selecting the optimal scales for every time interval, the pulse signal is reconstructed by inverse CWT. We denoted this method as “CWT-MAX” in the following.

**Fig. 2:**
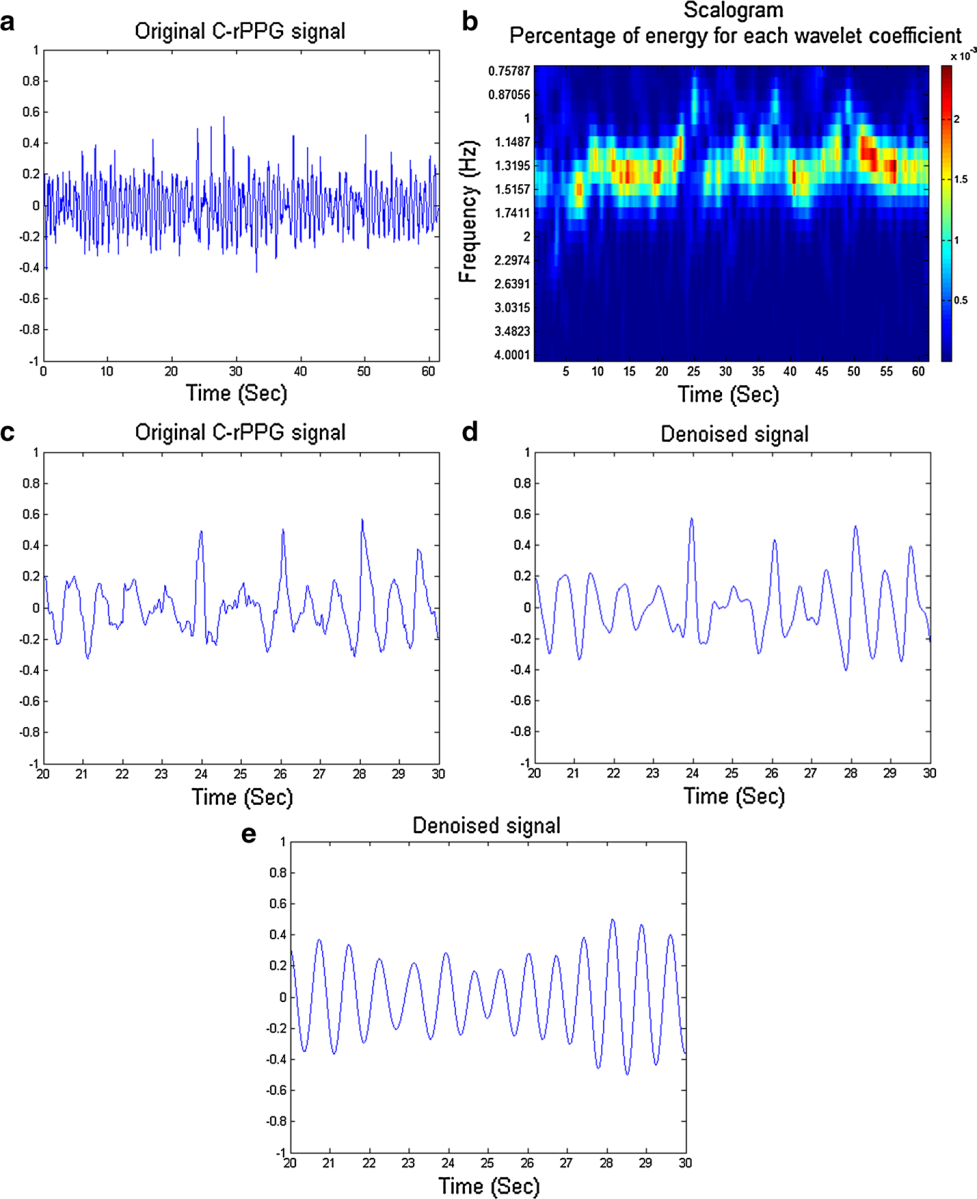
Examples for using CWT to detrend and denoise. **a** The original C-rPPG signal. **b** The CWT coefficients of the original signal. Note that the black solid line denotes the representative frequency (scales) of pulse signal computed by (13). **c** The zoomed-in part of original signal. **d** The zoomed-in part of signal denoised by CWT-BP. **e** The zoomed-in part of signal denoised by CWT-MAX

Figure 2 shows an example to demonstrate our approach. We can observe that the original signal shown in Fig. 2c is noisy and with many false peaks. After applying CWT to the original signal, we can obtain the CWT coefficients as shown in Fig. 2b. The *black line* represents the coefficients of optimal scales of every time interval. The CWT-BP reserved all the coefficients in the observed band [(0.75, 4) Hz] and set the others to zero which may smooth and denoise the original signal as shown in Fig. 2d; however, it still retained some false peaks which may degrade the accuracy of R–R intervals. On the contrary, the CWT-MAX only reserved the coefficients of representative scales of each time interval and set the others to zero. The signal reconstructed by CWT-MAX is much cleaner, as shown in Fig. 2e. Therefore, we applied the CWT-MAX in our algorithm.

### Peak detection and R–R intervals

**Fig. 3:**
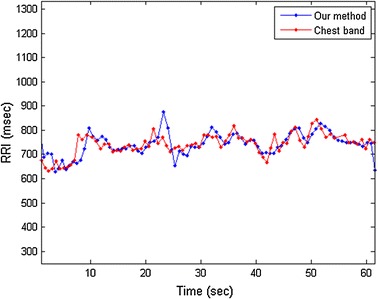
R–R intervals of the example in Fig. 2. The *red dot line* is the R–R intervals measured by an ECG-accurate chest band. The *blue dot line* is the R–R intervals computed by our method

After CWT denoising, the proposed approach then detect the peaks in the denoised pulse signal to compute the R–R intervals. One may simply use the *findpeaks* function built in MATLAB, or use the customized peak-finding algorithm. Since the CWT-denoised signal is almost noise-free, the selection of peak-finding algorithms does not play a crucial role to our results. After peak detection, let $p_k$ be the time instance of *k*-th peak in the signal, the R–R intervals can be calculated by

14 $$\begin{aligned} RRI_k = p_k-p_{k-1} \end{aligned}$$

and generally in the unit of millisecond (ms). Figure 3 shows the R–R intervals of the example in Fig. 2 computed by our method and the R–R intervals measured by an ECG-accurate chest band.

### Experimental setup

**Table 1:** Descriptions of the video clips in each category

Categories	Clip names	Descriptions
Static subjects	Static_1	The subject kept the body relaxed and static
	Static_2	There was desk light illuminated on the face
	Static_3	The subject kept smile during video recording
	Static_4	The subject kept making facial expression
Static subject with makeup	static_M1	The subject put the CC cream on her face
	Static_M2	The subject in static_M1 put additional powder foundation on her face
	Static_M3	The subject in static_M2 put additional blush powder on her face
Occasional motion	Motion_O1	The subject shook the head three times
	Motion_O2	The subject moved away from camera and then moved back
	Motion_O3	The subject turned the head, talking, then turned back, twice
Frequent motion	Motion_F1	The subject kept shaking his head
	Motion_F2	The subject rotated his head, move the body, or moved the camera several times

We totally taped 12 video clips with one minute long to evaluate the performance of R–R intervals extraction. The clips are taped by frontal camera of a smart phone (Sony Xperia Z1) with 30 fps frame rate and size of 640 × 480. We simulated the scenario that the video recorded the subjects when they were using their smart phone or tablet to monitoring their cardiac physiology. These video clips are classified into four categories, which are “static subjects”, “static subject with makeup”, “occasional motion”, and “frequent motion”, respectively. Each of the category has 2–4 clips with different subjects or slightly different conditions. Note that the word “static” here means the subjects kept their bodies static but slight movements (e.g., talking, facial expression, slight shaking) are allowable. The detail descriptions of the video clips are listed in the Table 1.

**Table 2:** Descriptions of all the subjects

Subjects	Genders	Ages	The corresponding video clips
Subject_1	Male	22	Static_1
Subject_2	Female	24	Static_2
Subject_3	Male	26	Static_3
Subject_4	Male	25	Static_4
Subject_5	Female	23	Static_M1, static_M2, and static_M3
Subject_6	Male	28	Motion_O1, motion_O2, motion_O3, motion_F1, and motion_F2

We have totally six subjects in the 22–28 age range involved in the experiments. There are four subjects (two males and two females) in “static subjects” category, one female subject in “static subject with makeup” category, and one male subject in both “occasional motion” and “frequent motion” categories, respectively. For more details of the subjects, please see the Table 2. The makeups we used in the experiments are, CC cream (CLINIQUE Molsture Surge CC cream hydrating colour corrector broad spectrum SPF30), powder foundation (DiorSnow Sublissime SPF30 PA+++), and blush powder (Christian Dior Diorshow Powder Backstage Makeup Color in a flash loose powder 0.17oz/5g 003 Catwalk Pink), respectively. This study had received approval by China Medical University and Hospital Research Ethics Committee. All the subjects have signed an informed consent allowing the authors to publish their HRV data.

We also used an ECG-accurate chest band (R1 Blue Comfortex+, made by Sigma sport) during video recording to obtain the ground truth of cardiac activity and exported the R–R intervals for the following comparisons. Because we aimed at the implementations suitable for smart phone applications, thus we only compared the proposed method with the algorithms which have similar computational cost. We will compare the performance of our algorithm with the ICA-based method [8] and original C-rPPG [10]. Since the authors did not release the source codes, we have tried our best to implement the algorithms described in their papers. We implemented the same bandpass filter in [8] for the original C-rPPG signal. For fair comparisons, we applied the same peak detection function (*findpeaks*) built in MATLAB to all the methods in the following experiments. Note that we do not further process the R–R intervals no matter they are reasonable or not. All the algorithms are implemented in MATLAB code.

## Results and discussions

### Quantitative evaluation

To show the accuracy of HRV estimation, this paper make comparisons with existed works by using well-known HRV metrics [26]. The scatter plot of R–R intervals is usually a good tool to show the relationship between $RRI_n$ and $RRI_{n+1}$ and thereby evaluate HRV. The scatter plot is a 2-D $(RRI_n, RRI_{n+1})$ plot, in which the calculated eigenvalues are useful in the following comparisons. The square root of an eigenvalue describes the standard deviation along the direction of corresponding eigenvector. In this paper we denote SD1 as the square root of the smallest eigenvalue and SD2 as the other one in our HRV comparisons. The time-domain HRV metrics used here are: the standard deviation of R–R intervals (SDNN), root mean square of successive differences (RMSSD), standard deviation of successive differences (SDSD). All the HRV metrics mentioned above are in the unit of millisecond (ms).

### Results and discussions of each category

#### Static subjects

**Table 3:** The HRV metrics estimated by different methods in the “static subjects” category

Clip names	HRV metrics (ms)	Chest band	ICA [8]		C-rPPG [10]		Our method	
		Est.	Est.	Abs. error	Est.	Abs. error	Est.	Abs. error
Static_1	SD1	22.02	68.89	46.87	61.77	39.75	24.89	2.87
	SD2	65.15	83.11	17.96	94.77	29.62	64.77	0.38
	SDNN	49.28	77.69	28.41	83.87	34.59	48.79	0.49
	RMSSD	30.81	96.31	65.50	87.30	56.49	34.63	3.82
	SDSD	31.21	97.88	66.67	88.59	57.38	35.20	3.99
Static_2	SD1	24.58	78.59	54.01	38.73	14.15	18.85	5.73
	SD2	72.80	90.66	17.86	79.03	6.23	69.40	3.40
	SDNN	54.03	85.56	31.53	62.35	8.32	50.75	3.28
	RMSSD	34.29	109.80	75.51	54.04	19.75	26.32	7.97
	SDSD	34.76	111.34	76.58	54.77	20.01	26.66	8.10
Static_3	SD1	22.37	89.13	66.76	62.31	39.94	18.79	3.58
	SD2	62.23	101.16	38.93	94.81	32.58	59.92	2.31
	SDNN	47.09	95.70	48.61	84.68	37.59	44.51	2.58
	RMSSD	31.25	140.83	109.58	87.59	56.34	26.16	5.09
	SDSD	31.67	143.04	111.37	88.92	57.25	26.54	5.13
Static_4	SD1	18.62	99.79	81.17	78.78	60.16	18.93	0.31
	SD2	38.41	107.31	68.90	91.64	53.23	41.15	2.74
	SDNN	30.21	104.91	74.70	86.29	56.08	31.91	1.70
	RMSSD	26.01	139.95	113.94	127.83	101.82	26.43	0.42
	SDSD	26.34	141.69	115.35	129.47	103.13	26.77	0.43

The HRV metrics of the “static subjects” clips estimated by the chest band and the different methods are listed in Table 3. Generally, the pulse signal extracted by C-rPPG [10] has better performance than the one extracted by ICA [8]. Our method inherited from C-rPPG and the HRV metrics are very close to the ones measured by chest band (see the absolute errors). Ideally, the clips with static subjects have no motion artifacts. However, as mentioned above, the HRV metrics are computed by R–R intervals which are very sensitive to the false peaks in the noisy signals. The proposed CWT-based denoising method removes the most interferences; hence, the R–R intervals are reliable and close to the ground truth even the subjects keep making facial expressions.

#### Static subjects with makeup

**Table 4:** The HRV metrics estimated by different methods in the “static subjects with makeup” category

Clip names	HRV metrics (ms)	Chest band	ICA [8]		C-rPPG [10]		Our method	
		Est.	Est.	Abs. error	Est.	Abs. error	Est.	Abs. error
Static_M1	SD1	14.95	101.16	86.21	97.89	82.94	19.59	4.64
	SD2	48.17	109.16	60.99	127.48	79.31	52.62	4.45
	SDNN	35.66	106.39	70.73	114.03	78.37	39.62	3.96
	RMSSD	20.85	141.85	121.00	136.41	115.56	27.28	6.43
	SDSD	21.14	143.76	122.62	138.52	117.38	27.71	6.57
Static_M2	SD1	13.97	49.93	35.96	54.71	40.74	13.94	0.03
	SD2	65.34	74.35	9.01	76.72	11.38	68.56	3.22
	SDNN	47.14	64.42	17.28	67.81	20.67	50.21	3.07
	RMSSD	19.50	69.77	50.27	76.45	56.95	19.59	0.09
	SDSD	19.75	70.77	51.02	77.52	57.77	19.72	0.03
Static_M3	SD1	20.96	71.56	50.6	79.62	58.66	23.95	2.99
	SD2	86.84	127.90	41.06	135.26	48.42	92.18	5.34
	SDNN	63.79	104.07	40.28	114.17	50.38	67.58	3.79
	RMSSD	29.34	99.66	70.32	111.30	81.96	33.43	4.09
	SDSD	29.73	101.20	71.47	112.88	83.15	33.92	4.19

In this category, we made experiments on the cases which the subjects had different kind of makeup on her face. Table 4 shows the results of HRV estimated by the different methods. The ICA-based method and C-rPPG deviated from the ground truth while our method still got much lower errors. In these experiments, we can observe that the makeup may interfere the performance of the pulse signal extraction. However, this interference will not degrade the results of our method because our technique can successfully remove noises and artifacts.

#### Occasional motion

**Fig. 4:**
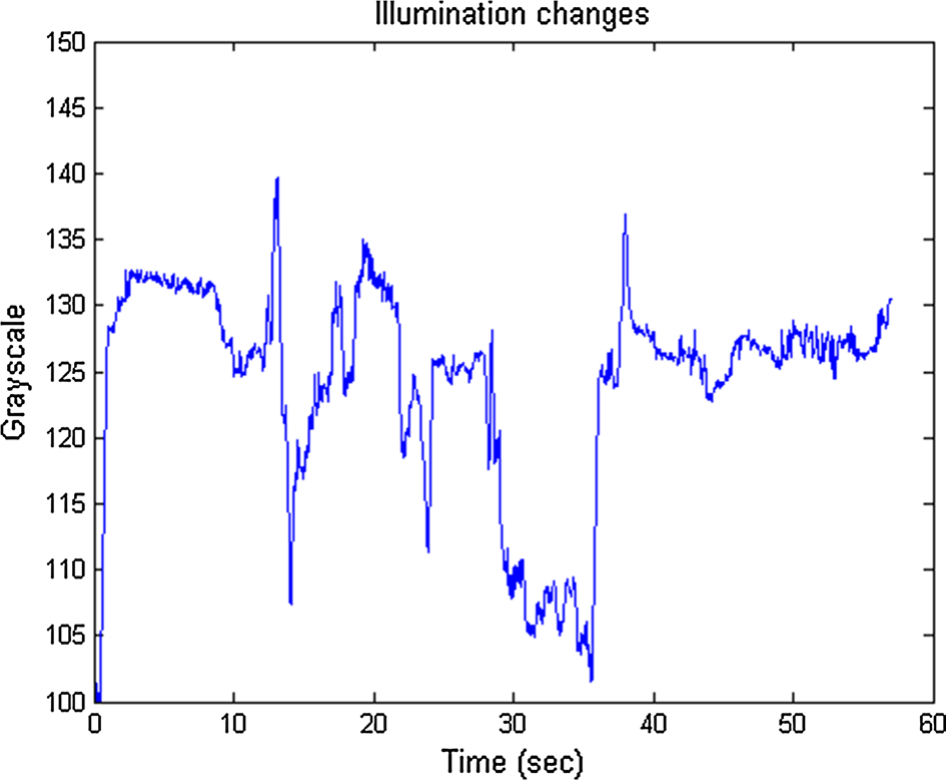
The illumination changes of the face in the “motion_O3” clip

**Table 5:** The HRV metrics estimated by different methods in the “occasional motion” category

Clip names	HRV metrics (ms)	Chest band	ICA [8]		C-rPPG [10]		Our method	
		Est.	Est.	Abs. error	Est.	Abs. error	Est.	Abs. error
Motion_O1	SD1	27.77	50.39	22.62	62.06	34.29	20.44	7.33
	SD2	59.82	68.47	8.65	89.43	29.61	56.38	3.44
	SDNN	46.94	60.03	13.09	78.82	31.88	42.08	4.86
	RMSSD	38.76	70.19	31.43	86.91	48.15	28.47	10.29
	SDSD	39.29	71.26	31.97	88.16	48.87	28.91	10.38
Motion_O2	SD1	20.84	87.95	67.11	65.91	45.07	29.20	8.36
	SD2	72.93	91.72	18.79	87.78	14.85	72.57	0.36
	SDNN	53.29	89.60	36.31	78.28	24.99	55.37	2.08
	RMSSD	29.07	122.65	93.58	91.99	62.92	40.74	11.67
	SDSD	29.47	124.39	94.92	93.26	63.79	41.31	11.84
Motion_O3	SD1	24.60	123.32	98.72	103.63	79.03	60.63	36.03
	SD2	62.16	206.86	144.70	152.90	90.74	90.54	28.38
	SDNN	47.63	170.02	122.39	131.73	84.10	76.50	28.87
	RMSSD	34.35	171.91	137.56	144.61	110.26	84.39	50.04
	SDSD	34.83	174.41	139.58	146.71	111.88	85.74	50.91

The clips in this category are that the subjects moved his/her body or head less than three times, just like the regular motions we make in daily-life. Table 5 shows the results of this category. Our method only severely deviated from the ground truth in the “motion_O3”. To explain the result, we computed the averaged illumination (grayscale) on the face of the “motion_O3”, as shown in Fig. 4. We found that the illumination changes significantly due to the auto-exposure function of camera. The camera changed the exposure automatically when the subject turned the head, while the other two clips (“motion_O1” and “motion_O2”) have no such illumination changes. Therefore, our method still obtained the HRV metrics close to the ground truth in “motion_O1” and “motion_O2”.

#### Frequent motion

**Fig. 5:**
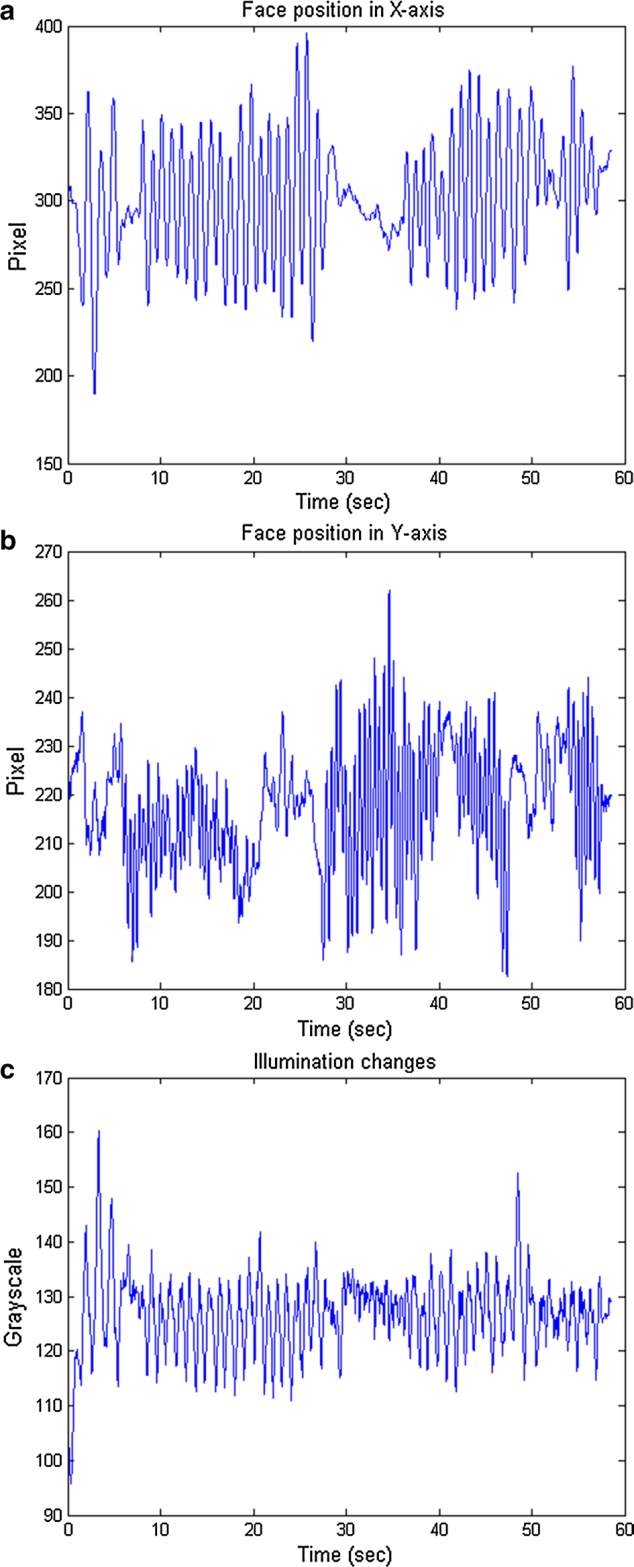
The face positions and illumination in “motion_F1” clip. **a** The face position (*x-axis*). **b** The face position (*y-axis*). **c** The illumination (grayscale) of the face

**Table 6:** The HRV metrics estimated by different methods in the “frequent motion” category

Clip names	HRV metrics (ms)	Chest band	ICA [8]		C-rPPG [10]		Our method	
		Est.	Est.	Abs. error	Est.	Abs. error	Est.	Abs. error
Motion_F1	SD1	19.78	177.67	157.89	89.05	69.27	27.05	7.27
	SD2	48.73	271.01	222.28	133.70	84.97	58.09	9.36
	SDNN	37.76	227.73	189.97	112.87	75.11	45.04	7.28
	RMSSD	27.72	247.65	219.93	124.22	96.50	37.72	10.00
	SDSD	28.03	251.26	223.23	125.94	97.91	38.26	10.23
Motion_F2	SD1	30.89	149.01	118.12	130.11	99.22	41.60	10.71
	SD2	95.04	255.18	160.14	164.69	69.65	99.60	4.56
	SDNN	71.15	207.28	136.13	147.28	76.13	75.97	4.82
	RMSSD	43.12	207.25	164.13	180.92	137.80	57.85	14.73
	SDSD	43.73	210.73	167.00	184.00	140.27	58.84	15.11

Table 6 shows the HRV metrics of the “frequent motion” video clips. This category is extremely challenging since the subjects kept making movements during the video recording. Both the ICA [8] and C-rPPG [10] severely deviated from the ground truth. Although our method was interfered by the large motion artifacts, we still obtained a reasonable HRV metrics close to ground truth. Figure 5 shows the face positions and the illumination changes on the face of “motion_F1”. The face positions changes periodically due to the continuously shaking of the head. We can observe that the illumination changed with the motion of face rather than exposure changes. The results have shown that our method can deal with the motion artifacts even the subject kept shaking his head during the video recording if the exposure of camera is almost fixed.

## Conclusion

In this paper, we have analyzed the problems of camera-based PPG and proposed an algorithm to extract accurate R–R intervals using 30 fps camera. We first extract the pulse signal using the chrominance-based method (C-rPPG) followed by a denoising method based on the CWT. The R–R intervals are computed by finding the peaks in the denoised signals. The experimental video clips were recorded by a frontal camera of smart phone (Sony Xperia Z1) held by the subjects in different situations. The experiments have shown that our method is able to extract much more accurate results than the related works. The mean of absolute errors of HRV metrics obtained by our method is only 3.53 ms in the “Static subjects” and “Static subjects with makeup” categories. This shows the potential of our method for remote health monitoring of patients, which can be done by an easy and comfortable way in daily-life.

Note that the measurements of HRV for clinical use should conform to professional recommendations (e.g., [1, 26]), and our method might not meet those requirements. However, it can be useful for informal applications; for instance, monitoring the physiological status of the tablet users and giving warnings to the users who may have some potential healthy problems.

Although the proposed method is able to alleviate the interference of motion artifacts, we still have room for improvement to deal with the artifacts made by the significantly changes of exposure due to auto-exposure function of camera. In addition, for a proof of concept, this paper validates our work with six subjects which might not be enough to show convincing, statistically significant evidence of efficacy. In the future, we will aim to refine our algorithm for being robust to artifacts generated by built-in functions in smart phones, and conduct the experiments with larger number of subjects.

## References

[1] Task Force of the European Society of Cardiology (1996). Heart rate variability standards of measurement, physiological interpretation, and clinical use. Eur Heart J.

[2] Hertzman AB (1937). Photoelectric plethysmography of the fingers and toes in man. Expl Biol Med.

[3] Peng R-C, Zhou X-L, Lin W-H, Zhang Y-T (2015). Extraction of heart rate variability from smartphone photoplethysmograms. Comput Math Methods Med.

[4] Huelsbusch M, Blazek V (2002). Contactless mapping of rhythmical phenomena in tissue perfusion using ppgi. Medical Imaging 2002.

[5] Takano C, Ohta Y (2007). Heart rate measurement based on a time-lapse image. Med Eng Phys.

[6] Verkruysse W, Svaasand LO, Nelson JS (2008). Remote plethysmographic imaging using ambient light. Opt Express.

[7] Poh M-Z, McDuff DJ, Picard RW (2010). Non-contact, automated cardiac pulse measurements using video imaging and blind source separation. Opt Express.

[8] Poh M-Z, McDuff DJ, Picard RW (2011). Advancements in noncontact, multiparameter physiological measurements using a webcam. IEEE Trans Biomed Eng.

[9] Lewandowska M, Rumiński J, Kocejko T, et al. Measuring pulse rate with a webcam-a non-contact method for evaluating cardiac activity. In: Computer science and information systems (FedCSIS), 2011 Federated Conference On. IEEE; 2011. p. 405–10.

[10] de Haan G, Jeanne V (2013). Robust pulse rate from chrominance-based rppg. IEEE Trans Biomed Eng.

[11] Wu H-Y, Rubinstein M, Shih E, Guttag JV, Durand F, Freeman WT (2012). Eulerian video magnification for revealing subtle changes in the world. ACM Trans Graph.

[12] Wang W, Stuijk S, de Haan G (2015). Exploiting spatial redundancy of image sensor for motion robust rppg. IEEE Trans Biomed Eng.

[13] Comon P (1994). Independent component analysis, a new concept?. Signal Process.

[14] Cardoso J-F (1999). High-order contrasts for independent component analysis. Neural Comput.

[15] Crowe JA, Damianou D. The wavelength dependence of the photoplethysmogram and its implication to pulse oximetry. In: Engineering in medicine and biology society, 1992 14th Annual International Conference of the IEEE, vol 6. IEEE; 1992. p. 2423–4.

[16] Martinez LFC, Paez G, Strojnik M. Optimal wavelength selection for noncontact reflection photoplethysmography. In: International Commission for Optics (ICO 22). Washington: International Society for Optics and Photonics; 2011. p. 801191.

[17] Tominaga S (1994). Dichromatic reflection models for a variety of materials. Color Res Appl.

[18] Viola P, Jones MJ (2004). Robust real-time face detection. Int J Comput Vis.

[19] Zhang C, Zhang Z. A survey of recent advances in face detection. Technical report : Tech. rep., Microsoft Research; 2010.

[20] Hjelmås E, Low BK (2001). Face detection: a survey. Comput Vis Image underst.

[21] Hsu R-L, Abdel-Mottaleb M, Jain AK (2002). Face detection in color images. Pattern Anal Mach Intel IEEE Trans.

[22] Vezhnevets V, Sazonov V, Andreeva A. A survey on pixel-based skin color detection techniques. In: Proc Graphicon, vol 3. Moscow: 2003. p. 85–92.

[23] Soni S, Namjoshi Y. Delineation of raw plethysmograph using wavelets for mobile based pulse oximeters. 2010. arXiv preprint arXiv:1011.0250.

[24] Peterek T, Prauzek M, Penhaker M. A new method for identification of the significant point in the plethysmografical record. In: Signal processing systems (ICSPS), 2010 2nd International Conference on, vol 1. IEEE; 2010. p. 1–362.

[25] Addison PS, Watson JN (2004). A novel time-frequency-based 3d lissajous figure method and its application to the determination of oxygen saturation from the photoplethysmogram. Meas Sci Technol.

[26] Stein P, Kleiger R (1999). MD: insights from the study of heart rate variability. Annu Rev Med.

